# Isolated Microcornea: Case Report and Relation to Other “Small Eye” Phenotypes

**DOI:** 10.4103/0974-9233.51999

**Published:** 2008

**Authors:** Ribhi Hazin, Arif O. Khan

**Affiliations:** 1 From Medical School Program, Harvard University, Boston, United States of America; 2 From Pediatric Ophthalmology Division, King Khaled Eye Specialist Hospital, Riyadh, Kingdom of Saudi Arabia

**Keywords:** isolated microcornea, relative anterior microphthalmos

## Abstract

Isolated microcornea is a very rarely-described condition of reduced cornea size (less than 11 mm horizontal diameter) in an individual without other significant ocular (or systemic) findings. This case report describes the biometric features of a boy with isolated microcornea (the youngest and most completely described patient to the best of our knowledge) and suggests that the condition is the same entity as relative anterior microphthalmos.

Isolated microcornea is a very rarely-described condition of reduced cornea size (less than 11 mm horizontal diameter) in an individual without other significant ocular (or systemic) findings.^1^–^2^ The purpose of this report is to describe the biometric features of a boy with isolated microcornea and to suggest that the condition is the same entity as relative anterior microphthalmos.^3^–^4^

## Case Report

A 12-year-old boy without other significant medical history was seen because of small eyes since birth (Figure). Neither his brother nor his sister had a history of ocular disease, and there was no family history of ocular or systemic disease. Visual acuity with his current glasses (-3.75-3.50×020 right eye [OD], -4.25-1.25×165 left eye [OS]) was 20/40 in both eyes. There was no strabismus and ductions were full. Pupillary exam was normal. Intraocular pressure by Goldmann tonometry was 14 mm Hg in either eye. Slit-lamp exam was significant for small but apparently otherwise normal corneas (10 mm horizontal diameter). The anterior chamber was otherwise within normal limits. Cycloplegic refraction (cyclopentolate 1%) was comparable to his current glasses. Retinal and optic disc examination by indirect ophthalmoloscopy (20 diopter [D] lens) was within normal limits. Anterior segment analysis was performed (Orbiscan IIZ, Bausch & Lomb, Salt Lake City, USA) and revealed the following: average central corneal radius of curvature of 7.6 mm OD and 7.5 mm OS (with 2 diopters of with-the-rule corneal astigmatism in both eyes), central corneal thickness of 610 microns OD and 591 microns OS, and anterior chamber depth of 2.70 mm OD and 2.86 mm OS. Axial lengths (IOLMaster, Zeiss Humphrey, Dublin, California, USA) were 23.57 mm OD and 23.61 mm OS. These results are summarized in the Table in the context of age-appropriate normal values.

**Figure:**
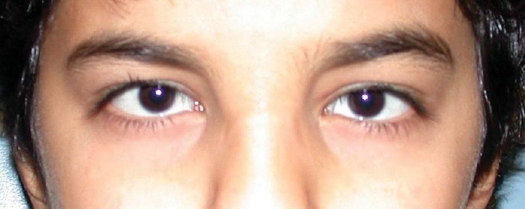
At 12 years of age, the child had horizontal corneal diameters of 10 mm but otherwise unremarkable anterior segments by slit-lamp examination.

## Discussion

Although it has been suggested that isolated microcornea (IM) occurs more commonly in short eyes,^5^ the few prior reports describe myopic eyes and do not include biometry.^1^,^2^ Biometry of our patient was significant for thick corneas and a short anterior chamber depths (Table). These findings suggest a primarily lenticular cause for our patient's myopia and lead us to believe that IM is the same as the more recently-described entity known as relative anterior microphthalmos (RAM).^3^,^4^ RAM has been used to describe eyes with reduced corneal diameter (<11 mm) and anterior segment depth in the setting of a normal axial length.^3^,^4^ To the best of our knowledge all previously-reported RAM cases have been in adults 36 years of age or older, patients who were at risk of pseudoexfoliation syndrome and glaucoma.^3^,^4^ IM/RAM may be due to an overgrowth of the anterior tips of the optic cup during embryologic development, resulting in minimal space for the cornea to develop.

**Table:** Patient Biometry at 12 Years of Age and Normal Ranges

	Our Patient	Normal Values
Corneal Central Thickness (microns)	610 OD, 591 OS	529 +/-34 (5-15 year olds)^9^

Anterior segment depth (mm)	2.70 OD, 2.86 OS	3.67 +/- 0.25 (12 year olds)^10^

Axial length (mm)	23.57 OD, 23.61 OS	23.38 +/-0.85 (12 year olds)^10^

Corneal Curvature (average, mm)	7.6 OD, 7.5 OS	7.78 +/- 0.25 (12 year olds)^10^

More common ‘small eye’ phenotypes include microphthalmos, nanophthalmos, posterior microphthalmos, and cornea plana. These conditions should be recognized as distinct entities and not be “lumped” together. Microphthalmos, an abnormally small globe, is a clinical spectrum of disease classified as either simple (without co-existent ocular defect) or complex (e.g., colobomatous, cataractous, with retinal detachment, syndromic).^5^ The prognosis of microphthalmic eyes depends upon the extent of coexisting ocular abnormality. Nanophthalmos refers to an abnormally-small eye with a normal-sized lens; biometry is needed to confirm the high lens/eye volume ratio.^6^ Nanophthalmic eyes have thickened posterior sclera and are prone to choridal effusions following intraocular surgery.^6^ Posterior microphthalmos refers to a foreshortened posterior segment with a normal-sized anterior segment; retinochoroidal folds suggest the diagnosis and ultrasound is diagnostic.^7^ These eyes may appear small even though the anterior segment is of normal dimension because of relative enophthalmos due to the short posterior segment. Cornea plana refers to an abnormally small and flat cornea; with the exception of occasional iris abnormalities affected eyes are typically otherwise normal.^8^ The recessive form is specific for homozygous *KERA* mutation.^8^ Unlike individuals with IM/RAM, children with microphthalmos, nanophthalmos, posterior micophthalmos, and cornea plana tend to have high hyperopia with related childhood complications of accommodative esotropia and amblyopia. All of these “small eye” phenotypes have a risk of glaucoma with the exception of posterior microphthalmos (in which the anterior segment is completely normal).

In summary, the rare entity of IM is likely the same condition as RAM. We encourage use of the more descriptive latter term to describe the findings of small cornea and short anterior segment depth in an otherwise healthy-eye. The various “small eye” phenotypes should be recognized as distinct conditions rather than be “lumped” together.
